# Validating and Extending the Three Process Model of Alertness in Airline Operations

**DOI:** 10.1371/journal.pone.0108679

**Published:** 2014-10-20

**Authors:** Michael Ingre, Wessel Van Leeuwen, Tomas Klemets, Christer Ullvetter, Stephen Hough, Göran Kecklund, David Karlsson, Torbjörn Åkerstedt

**Affiliations:** 1 Stress Research Institute, Stockholm University, Stockholm, Sweden; 2 Jeppesen Systems AB, Göteborg, Sweden; 3 Swedish Transport Agency, Norrköping, Sweden; 4 Scandinavian Airline Systems AB, Stockholm, Sweden; University of Alabama at Birmingham, United States of America

## Abstract

Sleepiness and fatigue are important risk factors in the transport sector and bio-mathematical sleepiness, sleep and fatigue modeling is increasingly becoming a valuable tool for assessing safety of work schedules and rosters in Fatigue Risk Management Systems (FRMS). The present study sought to validate the inner workings of one such model, Three Process Model (TPM), on aircrews and extend the model with functions to model jetlag and to directly assess the risk of any sleepiness level in any shift schedule or roster with and without knowledge of sleep timings. We collected sleep and sleepiness data from 136 aircrews in a real life situation by means of an application running on a handheld touch screen computer device (iPhone, iPod or iPad) and used the TPM to predict sleepiness with varying level of complexity of model equations and data. The results based on multilevel linear and non-linear mixed effects models showed that the TPM predictions correlated with observed ratings of sleepiness, but explorative analyses suggest that the default model can be improved and reduced to include only two-processes (S+C), with adjusted phases of the circadian process based on a single question of circadian type. We also extended the model with a function to model jetlag acclimatization and with estimates of individual differences including reference limits accounting for 50%, 75% and 90% of the population as well as functions for predicting the probability of any level of sleepiness for ecological assessment of absolute and relative risk of sleepiness in shift systems for safety applications.

## Introduction

Air transportation is the safest form of transportation per kilometer travelled [1], [2]. In the rare case of an accident, however, the results are often devastating. Accidents in aviation rarely have a single cause, and human errors are involved in the majority of them [3].

In road transport, the link between human error and fatigue has been established in several studies [4]. The main causes of sleepiness and fatigue are 1) circadian phase, 2) time awake, and 3) amount of prior sleep [5], [6]. In addition, time on task may induce fatigue when involving sustained attention [7]. Individual differences are likely to play a role in sleepiness and fatigue related accidents [8], driving performance [9], as well as modify sleep length [10] and performance during sleep deprivation [11]. Individual differences in the circadian type are among the most systematically studied with several rating scales developed to assess an approximate phase in individuals [12], [13].

Also in aviation, human errors and improper decision-making are influenced by sleepiness and fatigue [14]. Irregular working hours, working hours at inconvenient times of day, as well as frequent time zone crossings, characterize work life in aviation and all have a negative impact on alertness [15] and may increase the risk of accidents [16].

The problem of fatigue in pilots is almost as old as aviation itself [17]. It was, however, not until the 1980s that Samn and Perelli [18] developed a fatigue scale in order to subjectively measure fatigue levels in pilots, starting the investigation of the effect of multiple time zone crossings on pilot fatigue [19]. Since then, several factors have been shown to play a role in pilot fatigue and performance, including the highly automated work environment of the cockpit [20], flying at night [21], [22] as well as flight duration [23], [24], although it has been reported to be equally severe in short haul, as in long haul operations [25], [26].

One way of counteracting fatigue in aircrew is through flight and duty time limitations. However, regulatory bodies are currently discussing how to incorporate sleep and performance science directly into their fatigue risk management systems by means of bio-mathematical sleepiness and fatigue modeling [27]. Several such models have been introduced over the past decades [28]. Briefly, those include the two process model (2PM; [29]), the three process model of alertness (TPM, the subject of the present paper; [30]), the system for aircrew fatigue evaluation (SAFE; [31]), the interactive neurobehavioral model (INM; [32]), the sleep, activity, fatigue, and task effectiveness model (SAFTE; [33]), the fatigue audit inter dyne (FAID; [34]), and the circadian alertness simulator (CAS; [35]).

The key processes in those models (except FAID) include, although with different parameters: 1) a homeostatic process that describes the decline of alertness with time awake and its recovery with time asleep 2) a circadian process that describes the diurnal variation in alertness 3) a sleep inertia process that describes the delay after wake up before alertness resumes. In addition, some models estimate the decline of alertness with time on task (SAFE, FAID, and CAS). As the generated fatigue output, most models predict subjective alertness, except SAFTE (predicting performance effectiveness) and FAID (predicting violations based on risk threshold levels).

As Matschnigg et al. (2011) state (p10, chapter 4): “An important question to ask about any model is whether it has been validated against fatigue data from operations similar to those that you are interested in.” To our knowledge, the only model that has been extensively validated in many occupational settings is the TPM, though the present study is the first attempt to validate it on aircrew. Many shift work studies have shown accurate alertness predictions at the group level [36]–[39]. Although SAFE and SAFTE are specifically developed for use in aviation, those models still lack peer-reviewed validation.

Since its inception in 1990, the TPM has been extended with an extra component modeling a 12 h ultradian process [40] and a “brake” function that modifies homeostatic recovery during sleep [41]. The added predictive power of these modifications have, however, not been properly validated on empirical data. In addition, the TPM has also used several different linear transfer functions between the internal alertness score and empirical data using the Karolinska Sleepiness Scale that may give very different levels of sleepiness as the output. The TPM has also been extended with a model based sleep generator that can be used to insert sleep periods into the data when such data is not available [38], [40]. This sleep generator has been shown to predict sleep reasonably well in one specific compressed shift sequence [40] but has otherwise not been validated.

### Objectives

A main objective of the present study was to ***validate*** the TPM on a group of aircrewin real life situations, using observed sleep and sleepiness data:

- Validate the predictive power of all individual components and the “brake” function
- Validate the model based generated sleeps
- Estimate the best linear transformation function between observed data and model

Our second objective with the present study was to ***extend*** the model with estimates of individual differences and probability of sleepiness for ecological estimates of risk:

- Estimate probabilities of any level of sleepiness
- Estimate the influence of individual differences over and above the predicted group mean/median and calculate reference limits accounting for 50, 75 and 90% of the individuals

The circadian system is a large source of individual differences that may be of particular importance for aircrews that often travel across several time zones and become exposed to jetlag. Our third objective was to ***explore*** the feasibility of adjustment of the circadian phase according to circadian type and acclimatization to a different time zone for improved predictions of aircrews:

- Can we improve predictions by adjusting the phase of the circadian process based on a simple circadian type question?
- Can we improve the predictions after travelling to a different time zone by gradually adjusting the phase of the circadian process?

## Methods

### Subjects and procedure

Data collection took place in three waves over a two year time period (in the years 2011–2012) using a crowd sourcing strategy where interested aircrew signed up for participation in the study at the website of Jeppesen AB. Data was collected by means of an application (CrewAlert lite) running on a handheld touch screen computer/phone device (Apple: iPhone, iPod or iPad) and was submitted wirelessly over the Internet to a database at Jeppesen AB.

Participants for the study were recruited via the Jeppesen website, announcements of the upcoming data collection at conferences, and a number of airlines and unions in turn forwarding information to their crew. Crew was directed to a web page where they signed up and was sent e-learning material for self-studies that consisted of a document as well as a series of video clips instructing how to use the device for data collection. Briefly, participants were carefully instructed on how to enter their working times, sleep times and how to rate their sleepiness levels using the application. They could rate sleepiness at any time but, instructions were also included to provide ratings at top of climb, top of descent and whenever feeling sleepy. Crew also received a survey-code (hidden inside the information material) to be used when uploading the data. This provided some feedback that the participants had watched the videos and a possibility of knowing from which airline their data originated.

The application used to enter and submit data was equipped with a convenience function that would suggest a sleep timing for the user to confirm, or insert an automatically generated sleep if it was completely missing, based on a proprietary algorithm. This convenience function was always active unless the user had created a custom sleep log for a specific time period where the timing of sleeps was forced to be entered manually. To avoid circular analyses and ensure that data was entered independently of any sleep/sleepiness algorithm, we only selected data included in such custom sleep logs for further analysis. Descriptive statistics of the subjects participating in this study is presented for the whole sample and the restricted sample in table 1.

**Table 1:** Descriptives.

Variable	Restricted sample		Full sample
	Home base time zone	Any time zone	
Subjects (n)	130	136	153
Age (mean years)	42	41	41
Age (sd years)	8	8	8
Gender (% males)	92%	91%	94%
Position			
Captain	50%	49%	50%
First Officer	42%	42%	41%
Cabin crew	4%	4%	4%
Other	5%	4%	5%
Diurnal type			
Extreme evening	2%	1%	1%
Evening	26%	26%	25%
Intermediate	44%	45%	45%
Morning	28%	27%	28%

### Ethics statement

The participants were promised no other compensation than a report of the aggregated results, and a small chance to win a hand held touch screen computer (iPad) in a raffle among the half of crew providing the most valuable data (a weighting on the amount of days and data points provided). Since all contact with the participants where made electronically over the Internet, traditional written consent was not feasible to obtain and participants indicated informed consent to participate in the study by ticking a check-box in the application (CrewAlert lite) used for collecting and submitting the data. Participants were also required to include a survey code hidden inside the information material giving some confidence that they were well informed about the study. The study was approved by the regional ethical review board of the Gothenburg region (“Regionala etiskprövningsnämnden i Göteborg”).

### Variables

The application used to enter data collected numerous variables related to work, airline operations and sleep/sleepiness. These included personal (i.e., between subject) variables: circadian type, habitual sleep length, sleep quality in general, sleep termination, snoring, melatonin and coffee use, gender, birth year, height, weight, work position. It also included situational (i.e. within subject) variables: describing duty and sleep periods, sleepiness [42], fatigue [18] and a five minute test of psychomotor vigilance [43]. Number of flights within the duty was entered, as well as the type of duty (flight, dead head flight (as passenger), simulator check, simulator instructing, standby, ground duty, or off duty). Finally, the duration of briefing and debriefing were entered, as well as the duration of in-flight sleep.

For the purpose of the present study, we only used a subset of these variables described in more detail below:

We reported age (based on the indicated birth year), gender and work position (Captain, first officer, cabin crew and other) as descriptive statistics in table 1.

Circadian type was measured on a five point scale (Extreme morning type, morning type, intermediate type, evening type, extreme evening type). Instructions on how to assess your own type (based on preferred sleep timing) was included in the instructional videos.

For every duty (work shift), the first departure and last arrival airport was entered as well as the corresponding times of first departure and last arrival and the corresponding time zones. Sleep/wake behaviour was entered by adding a sleep/wake log period. Within those periods sleep start and end times were added by the user.

The main variable in TPM is sleepiness, and it could be rated at any time using the Karolinska Sleepiness Scale (KSS, 1 = “Extremely alert”, 2 = “Very alert”, 3 = “Alert”, 4 = “Rather alert”, 5 = “Neither alert nor sleepy”, 6 = “Some signs of sleepiness”, 7 = “Sleepy, but no effort to keep awake”, 8 = “Sleepy, some effort to keep awake”, 9 = “Very sleepy, great effort to keep awake, fighting sleep” [42]).

### Data quality validation

The software application (CrewAlert) and procedure to collect data featured advanced validation procedures to ensure the veracity of the collected data:

- Work duties could only be entered in a consistent way (departure before arrival, not overlapping each other, consistent time-zone transitions). Too long/short duties was also easily spotted at entry by the user through the graphical representation on screen.
- All three possible sleep-states were registered by the software: confirmed wake, confirmed sleep, and unknown state. (typical design was to assume wakefulness if sleep was non-present.). Sleep/wake logs were made impossible to overlap each other and sleep periods outside sleep/wake logs could not be entered.
- The user was taken directly to the data input screen when the application was activated to encourage data submission and shield the user from prior rating history to reduce any bias due to anchoring.
- All data was manually inspected and uploads that were missing both sleep/wake logs and sleepiness assessments were discarded. The discarded data sets, presumably from crew unwilling to follow-through on the data collection, constituted less than 3% of the total number of uploads. Any upload submitted without a valid survey code (see above: subjects and procedure) was ignored.

### The model equations

The main model parameters were estimated earlier [30] and have been published before [40], [44] but are briefly described below.

Two constants are used in the equations describing the higher (ha = 14.3) and lower (la = 2.4) asymptote of the internal alertness scale for the homeostatic process that describe decline in alertness as a function of wakefulness and recovery in alertness during sleep. During wake, this process is called S (1.1). It also takes as input the level of S at the time of awaking (sw) and calculates the decay in alertness (d = -0.0353) as a function of time awake (taw).
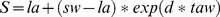
(1.1)


During sleep there are three different functions describing the homeostatic process. The first one is called S’ (1.2) and represents the original process from 1990. It takes the level of S at the time of falling asleep (ss) as input. Process S’ was later modified with a “brake function” that split the process into S’_1_ (1.3) for the part of sleep with high homeostatic pressure defined by (S’<bl = 12.2) and S’_2_ (1.4) for the last part of sleep with lower pressure. These functions take another parameter (g =  log((ha −14.0)/(ha - 7.96))/8≈-0.3813) to calculate the recovery of alertness as a function of time asleep (tas) and use a calculation of the brake point in time asleep (bt) to decide when there is a switch from S’_1_ to S’_2_ (1.5).



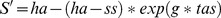
(1.2)

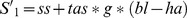
(1.3)

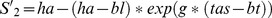
(1.4)

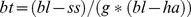
(1.5)


The model also includes a sleep inertia function (process W) that initially reduces alertness at the time of waking up (Wc = -5.72) with an exponential recovery (Wd = -1.51) as a function of time awake (taw).
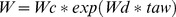
(1.6)


There are two processes related to time of day (tod). Process C (1.7) has a period of 24 h with a default circadian phase (p = 16.8), amplitude (Ca = 2.5) and mesor (Cm = 0). Process U (1.8) has a period of 12 h with amplitude (Ua = .5) and a mesor (Um = -.5).
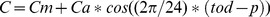
(1.7)

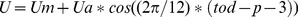
(1.8)


The model components are added together to produce a combined alertness score that can be used to predict ratings on the Karolinska Sleepiness Scale (KSS) using a linear transformation function with one constant (a = 10.6) and one coefficient (b = -.6) as described in equation 1.9 for the full model.
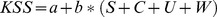
(1.9)


The model also includes a sleep generator that may be used to automatically insert sleep in data when no observed sleep is available. The sleep generator is part of the legacy of TPM and is in its current implementation based on the alertness score as well as the proximity to duty and sleep periods. The threshold for falling asleep is set by default to S+C+U<8.38 and the threshold for waking up is S+C+U>11.38. There is no sleep allowed within ±1 hour of a duty period and no sleep earlier than 2 hours after waking up. Waking up will not occur during the first hour of sleep. These restrictions were included to prevent flip/flop behavior of repeatedly falling asleep and waking up in certain situations.

One objective was to test the hypothesis that acclimatization can be made to different time zones by adjusting the phase of C (and U). For this purpose we developed a new process “A” to keep track of the acclimatized differences between the internal circadian phase and home base time used by the model. We modeled acclimatization to be made continuously, and to test our hypothesis we assumed a default daily rate of half the difference (daily = 50%) between the acclimatized time and local time as suggested by [45]. The equation is presented below (1.10) with “T” describing time in days and “TZ” describing the difference between local time and home base time and subscript describing measurement points in time (t). Acclimatized predictions from the model are obtained by adding A to the phase parameter (p) of process C and U.
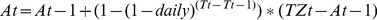
(1.10)


### Data selection and model predictions

**Figure 1:**
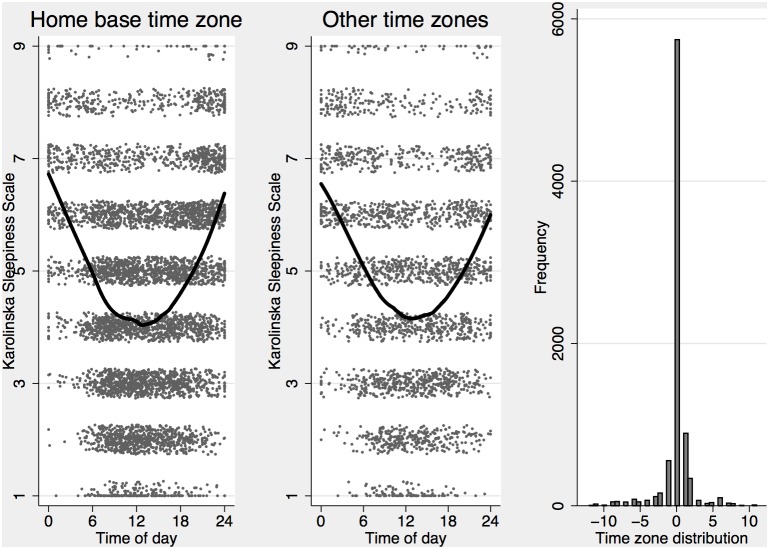
Observed ratings of subjective sleepiness on the Karolinska Sleepiness Scale (KSS) plotted against time of day (at home base) with a LOWESS line indicating approximate means for home base time zone (left) and other time zones (middle) with the overall distribution of time zones for all ratings (right). KSS ratings have had a random jitter applied to better illustrate the distribution at different levels.

We implemented the equations for the alertness score and model based generated sleep times in the R programming language [46] and used the first sleep entered into the sleep logs as a start for our algorithm, assuming that individuals fell asleep at the level of alertness used by the default sleep generator (S+C+U = 8.38). While such assumption is reasonable in many situations, it will not accurately account for a complex sleep history. To give the model reasonably accurate starting values we only analyzed sleepiness data where there was a sleep history of at least two sleeps. We also included all sleepiness ratings up until 16 h after the last logged sleep had ended. This provided us with 964 sleep logs containing 5443 sleeps (3515 after the second sleep) for 136 subjects and a total of 8040 ratings of sleepiness available for analysis. Of these, 2296 ratings occurred in a time zone different from the home base, leaving 5744 observations for the main analysis. Sleepiness ratings plotted against time of day at home base time zone and other time zones are presented in figure 1.

**Figure 2:**
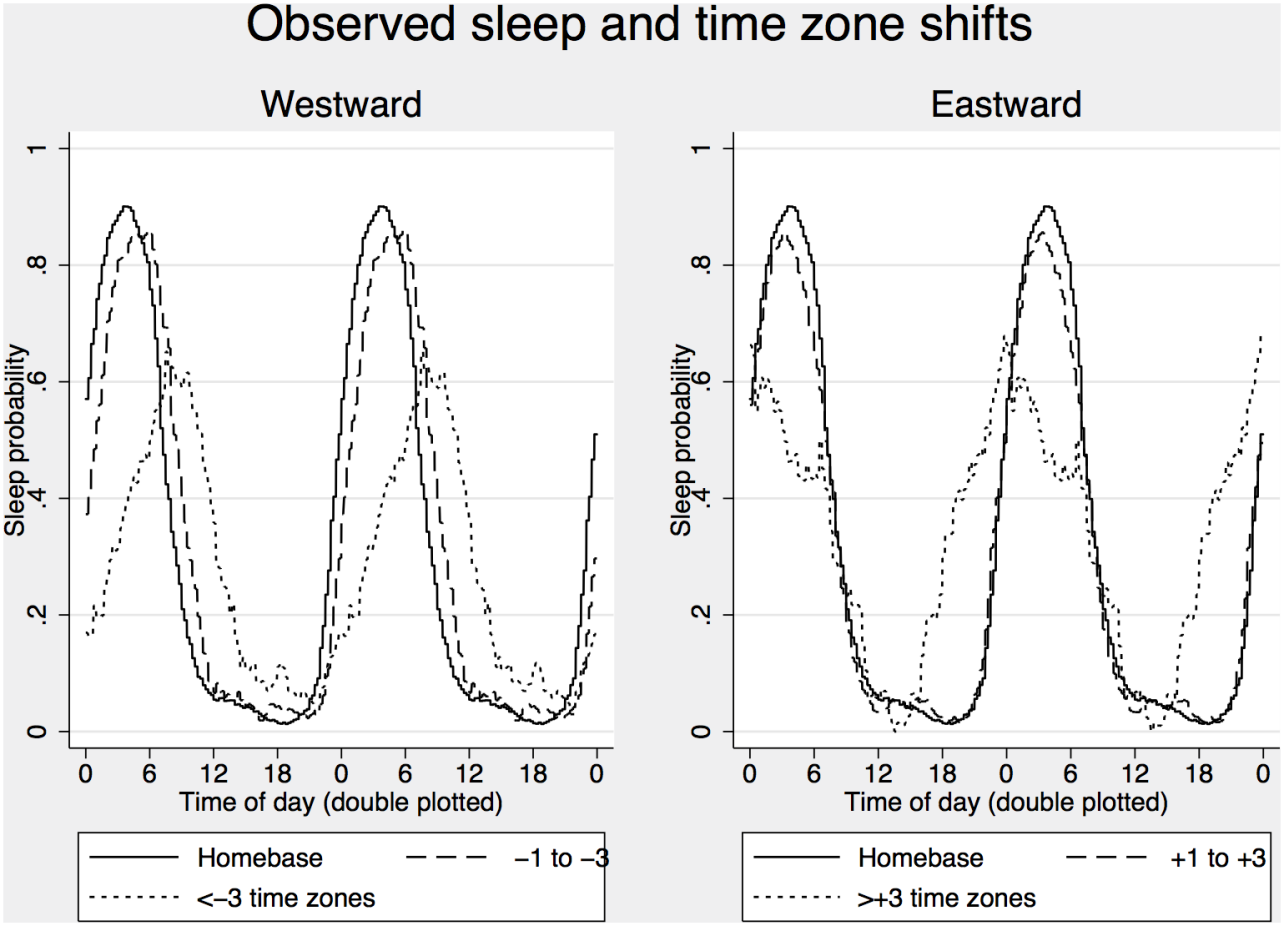
Observed sleep probability across time of day for the home base time zone and westward time zone shifts (left) and eastward time zone shifts (right).

The purpose of the sleep generator is to enable predictions when there is no information of sleeps available. However, to make the data comparable with the analysis of sleepiness ratings described above we used the first observed sleep in the log as a starting point for the algorithm. This was based on the assumption that the sleep started at the default alertness score used by the sleep generator (S+C+U = 8.38) and ended at the hour indicated in the log, and excluded it from subsequent analysis. We generated a dataset with observed and generated sleep/wake status for every 5-minute interval over the time period covered by the sleep log for every individual for further analysis of sleep propensity across time of day. The distribution of sleep propensity across time of day is presented in figure 2.

### Statistical analyses

To validate the individual components of TPM we fitted multilevel mixed effect linear regression models with alertness score (alert) as the independent variable and observed subjective sleepiness ratings (KSS) as the dependent variable. The model also included random intercepts to account for systematic variation in sleepiness propensity between subjects. A linear equation describing the model with regression parameters (β), latent variables/random effects (η) and residual error (ε) including subscript for individual observations (i) and subjects (j) is presented below (1.11). The random intercept variance was used to calculate reference limits accounting for 75% and 90% of the individuals in addition to the mean (50% of individuals in the group).
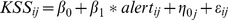
(1.11)


To estimate the optimal phase for process C in our data we started with a simple alertness score from process S only (to remove the effect of time awake on data), and added a standard cosinor analysis to the model, with independent variables describing a 24 h period sine (sin) and cosine (cos) function of time of day (1.12). The phase was subsequently calculated as (ARCTAN(β_1_/β_2_)*24)/2π + K+12, where K = 0, 12, 12 or 24 depending on the sign of the coefficients β_1_/β_2_ (+/+, +/−, −/− or −/+), and the amplitude was calculated as sqrt(β_1_∧2+ β_2_∧2). To estimate individual phases for all subjects we added random effects for the sine and cosine functions and used the model to predict empirical Bayes’ estimates of individual phases (1.13).
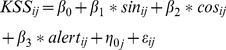
(1.12)

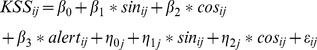
(1.13)


The empirical Bayes’ estimates of individual phases were subjected to a standard cross-sectional linear regression model with one observation for each subject and circadian type as the independent variable to get a predicted phase adjustment for process C (and U) based on the circadian type question.

The model so far has been developed using linear statistical models. And while such an approach is reasonable in many situations, one cannot exclude a possible bias in the estimates if the scale used to collect data (i.e. KSS) would violate some assumptions behind linear models. For example, estimates could become biased if the distances between categories on the scale differ systematically so they are longer in one end of the scale. To address potential concerns with linear models and to provide direct estimates of probabilities instead of group means, we used a generalized multilevel mixed model to fit an ordered (proportional odds) logistic regression on data. The model is a generalization of the linear model where the probability of observing outcome k or higher depends on the linear predictor *xb* (i.e. the sum of the right hand side of equations 1.11–1.13 excluding the error term ε_ij_) and a set of parameters defining cut points (K) between all levels of the scale of the dependent variable (1.14). The model is reduced to a binary logistic regression model when there are no cut points (K) to be estimated, and such equation was used to predict the probability of observing sleep (sleep) as a function of automatically generated sleep (xb_ij_ = β_0_+β_1_*generated sleep_ij_+η_0j_) presented in equation 1.15.
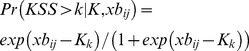
(1.14)

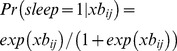
(1.15)


Model testing was performed in a hierarchical way starting with a null model, fitting only a constant as well as a baseline model fitting a constant and a random intercept, to estimate the raw mean and variance in data and then adding processes and complexity step by step.

Most models fitted used equation 11 with a single parameter describing the combined alertness score. This means that they technically have the same number of parameters and that differences in goodness of fit can be assessed in the log likelihood. However, such an approach does not take into account the complexity of the alertness score calculations that can be the result of anything from one to more than ten equations with varying number of parameters (assumed to be fixed and constrained to a known value) and varying data requirements (e.g. observed sleep, time-zones and circadian type).

To assess increase in model fit between two models using equation 11 above, an approximate likelihood ratio testing procedure was applied using a deviance statistic calculated as 2*-log likelihood difference between two (nested) models. This statistic can be used for likelihood ratio testing of two nested models with a chi2 distribution and degrees of freedom equal to the difference in the number of parameters between two nested models. For the purpose of penalizing increased complexity by adding a process to the model (S, C, U, W, A), a modification to a process (i.e. the “brake function” modifying process S) or adjusting the circadian phase of C and U based on the circadian type question, we assumed 2 degrees of freedom for each step giving a critical deviance chi2≈6 for approximate likelihood ratio testing with α = .05.

All statistical analyses were performed using Stata 13 with the procedures *mixed* and *meologit* to fit the models and *predict* to generate empirical Bayes’ estimates [47].

## Results

A summary of the models fitted is presented in table 2. Overall model fit is summarized in the log likelihood statistics, the residual (ε) and subject level random effect (η) standard deviations, but selected deviance statistics (2*−log likelihood difference between two models) are also presented for approximate likelihood ratio testing. Assuming a difference in complexity between two nested models of df≈2 would suggest a chi2>6 to provide significant (p<.05) improvement of model fit.

**Table 2:** Model fitting summary.

Model		Goodness of fit				Deviance		Transfer to KSS	
No	Description	log L	ε		η	vs	chi2	a	b
	*Models with observed sleeps in the home base time zone (n = 5744)*								
1a	Null model	−11798.9		1.887				4.58	
1b	Random intercept	−11174.1		1.656	0.809	1a	1250	4.62	
2a	C	−11125.7		1.642	0.813	1b	97	4.67	−0.14
2b	S	−10559.8		1.485	0.808	1b	1229	9.82	−0.45
2c	S_B_	−10520.5		1.474	0.828	2b	79	9.70	−0.46
3a	SC	−10315.0		1.422	0.819	1b	1718	10.19	−0.47
3b	SCW	−10629.3		1.504	0.815	3a	−629	8.51	−0.35
3c	SCU	−10298.2		1.418	0.818	3a	34	10.03	−0.48
3d	SCUW	−10590.4		1.493	0.814	3c	−584	8.57	−0.36
4a	SC_p15h_	−10151.9		1.382	0.810			9.46	−0.40
4b	SC_p15_U	−10163.8		1.385	0.809			9.13	−0.39
4c	SC_T_U	−10114.0		1.372	0.811			9.41	−0.42
4d	SC_T_	−10102.6		1.370	0.811	4c	23	9.75	−0.43
5a	S_B_C	−10313.8		1.421	0.838	3a	2	9.83	−0.46
5b	S_B_CW	−10629.5		1.503	0.823	5a	−631	8.24	−0.33
*5c*	S_B_CU	−10297.7		1.417	0.839	5a	32	9.68	−0.46
*5d*	S_B_CUW	−10593.0		1.494	0.823	5c	−591	8.28	−0.35
*6a*	S_B_C_p15h_	−10118.6		1.373	0.827	5c	358	9.30	−0.41
*6b*	S_B_C_p15h_U	−10128.0		1.375	0.826	5c	339	8.99	−0.40
*6c*	S_B_C_T_U	−10083.5		1.364	0.829	5c	428	9.24	−0.42
*6d*	S_B_C_T_	−10074.5		1.362	0.829	5c	446	9.56	−0.43
	*Models with generated sleeps in the home base time zone (n = 5744)*								
*7a*	S_B_CU	−10464.8		1.460	0.826	5c	−334	9.68	−0.47
*7b*	S_B_C_p15h_U	−10468.2		1.461	0.812	7a	−7	9.03	−0.42
*7c*	S_B_C_p15h_U (new thresholds)	−10279.4		1.413	0.809	7a	371	8.71	−0.37
*7d*	S_B_C_T_U (new thresholds)	−10245.1		1.405	0.812	7a	439	9.04	−0.40
*8a*	S_B_C	−10488.8		1.466	0.822	7a	−48	9.77	−0.46
*8b*	S_B_C_p15h_	−10477.6		1.464	0.811	7a	−26	9.30	−0.42
*8c*	S_B_C_p15h_ (new thresholds)	−10273.7		1.412	0.808	7a	382	8.99	−0.38
*8d*	S_B_C_T_ (new thresholds)	−10241.6		1.404	0.811	7a	446	9.33	−0.40
	*Models on data in all time zones (n = 8040)*								
*10a*	S_B_C	−15344.6		1.449	0.816			9.74	−0.45
*10b*	S_B_C_p15h_	−15081.1		1.404	0.807	10a	527	9.30	−0.41
*10c*	S_B_C_T_	−15027.5		1.395	0.807	10a	634	9.52	−0.42
*11a*	S_B_C_T_A_100%_	−15086.0		1.405	0.806	10c	−117	9.49	−0.42
*11b*	S_B_C_T_A_50%_	−15003.8		1.391	0.806	10c	47	9.54	−0.43
11c	S_B_C_T_A_30%_	−14999.1		1.391	0.806	10c	57	9.55	−0.43

Note. Description lists model components (SCUWA) used to calculate the alertness score. Subscripts indicate the presence of the “brake function” described in equation 2–3 for process S (B), if the phase of C was different from the default p = 16.8 h based on circadian type (T) or set at 15 h (p15h) for all subjects, and the daily acclimatization rate for process A (%). log L = log likelihood. ε = residual standard deviation. η = subject level random effect standard deviation. All models (except 1a & 1b) are based on equation 11 and differ only in how the alertness score was calculated. Deviance indicate 2*- log likelihood difference (chi2) compared to selected models (vs). Transfer to KSS describes the best fitting constant (a) and coefficient (b) to transfer the alertness score to KSS (equation 9).

### Validating the TPM

There was a large increase in log likelihood (deviance chi2>97) from the baseline random intercept model (1b) when process S (2a) and S+C (3b) were added to the model, suggesting at least the two processes S and C (3b) as a candidate model with a deviance chi2 = 1718 compared to the baseline model (1b).

The “brake” function modifying process S_B_ (2c) showed better fit than process S without the brake (2b) with a deviance of chi2 = 79 (p<.001). The brake was further evaluated in more complex models comparing models without the brake (models 3–4, table 2) to models with the brake (models 5–6, table 2) suggesting increased fit and a highly significant deviance in favor of the “brake” function comparing the best fitted models (6d & 4d) (chi2 = 67, df≈2, p<.001).

Expanding the model further by adding process W *decreased* goodness of fit (5b & 5d) but adding the ultradian process U increased fit (5c) with a significant deviance statistic (chi2 = 32, df≈2, p = .001).

**Figure 3:**
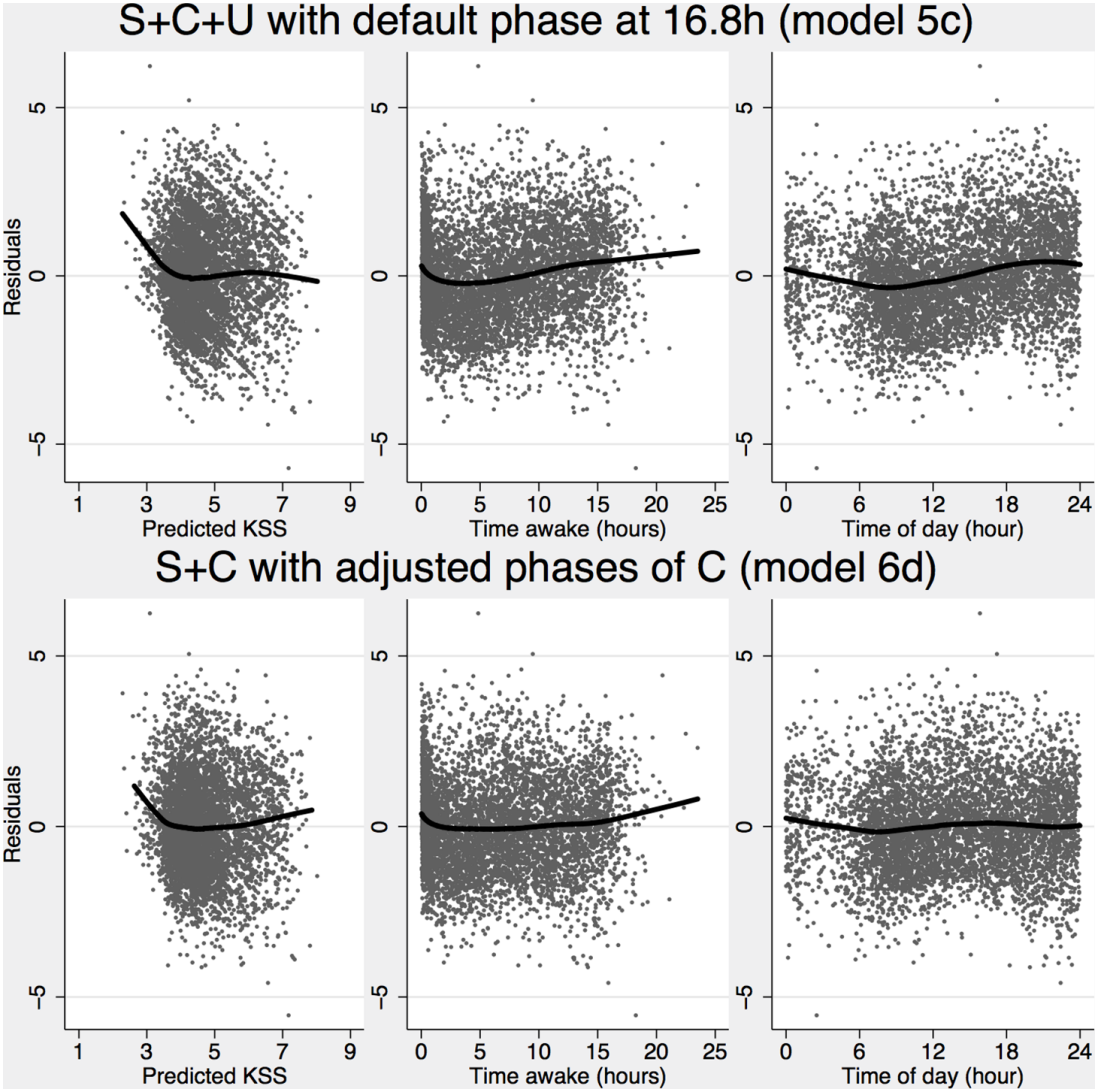
Residuals (observed ratings-predicted sleepiness) plotted against predicted KSS (left), time awake (middle) and time of day (right) for the best model with assumed default phase of C (model 5c, top) and circadian type adjusted phases of C_T_ (model 6d, bottom) with a LOWESS line indicating potential systematic bias in predictions.

The validation suggests that the optimal model for predicting sleepiness is based on process S_B_+C+U (model 5c). The optimal transfer function between the alertness score and observed KSS includes a constant (a = 9.68) and a coefficient (b = −.46) multiplying the alertness score as described in equation 9 with a residual error standard deviation of 1.42 and systematic variation between subjects over the intercept with a standard deviation of.84. Residual plots presented in figure 3 (top panels) suggest that there might be some systematic bias in the model at very high and very low levels of predicted sleepiness, but data is sparse at the ends of the scale. There is some evidence of a systematic bias related to time awake and time of day, suggesting that process S, C and U may not be optimally estimated, but the magnitude of this bias is relatively small. There was a small but clearly visible deviation during the first hour awake consistent with sleep inertia. However, sleep inertia as modeled by process W, provided a much worse fit when added to the model, suggesting that the default parameters of process W were exaggerated.

**Figure 4:**
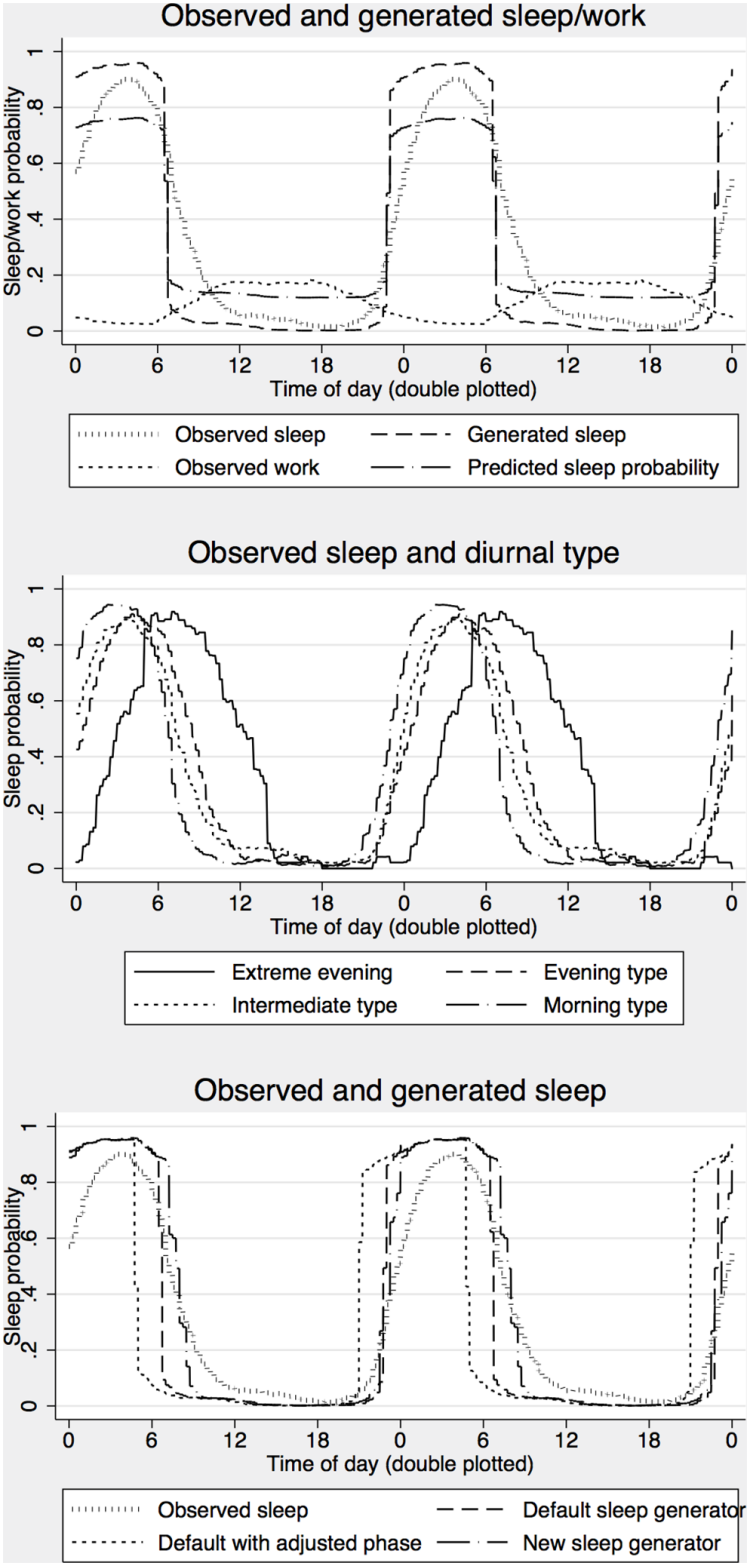
Observed, predicted and generated sleep. The **top panel** shows the proportion of observed 5 minute segments (n = 577969 for 118 subjects with sleep data) with observed duty, observed sleep, sleep generated by the TPM (with default thresholds) and predicted sleep based on the fixed part of a multilevel mixed effects logistic regression (equation 15) with observed sleep as the dependent variable and generated sleep as the predictor. The **middle panel** shows observed sleep for different circadian types. The **bottom panel** shows the output from three different sleep generators. The default sleep generator used a fixed phase of C (p = 16.8) for all subjects and threshold of S+C+U at 8.38 for falling asleep and 11.38 for waking up. Default sleep generator with adjusted phase use individually adjusted phases (p = 14.61 h, 15.28 h, 15.95 h, 16.62 h) depending on rated circadian type (morning type – extreme evening type). The new sleep generator use adjusted phases of C_T_ with a threshold for falling asleep of S+C<8 and waking up S+C>13.

We also wanted to validate the model based sleep generator and used it to generate sleep and wake status for all individuals and five-minute segments in data. The generated sleep was submitted to a multilevel mixed effect logistic regression model (equation 1.15) with observed sleep as the dependent variable for all observed five-minute time segments (n = 577969) and 118 subjects with observed sleep data. The result suggests a conditional probability of being asleep at pr = .118 when there was no sleep generated, with an increase to pr = .790 when there was sleep generated. The odds ratio of observing a sleep when the TPM had one generated was estimated at OR = 28 (95% CI: 27.6–28.4). Observed, generated (TPM) and predicted sleep probability (based on the logistic model), together with observed work probability is presented in figure 4 (top panels).

When model based generated sleeps were used instead of observed sleeps (7a) the residual error in the prediction increased to ε = 1.46. The deviance statistics comparing the model with observed sleeps (5c) was relatively large (chi2≈334), suggesting that access to observed sleep will increase the accuracy of the predictions.

### Extending the model with probabilities, individual differences and reference limits

Based on the estimates of model 5c we can extend the linear transformation function presented in equation 1.9 to also include individual differences and reference limits accounting for 90% and 75% of the subjects in addition to the group mean. The function is presented below with the offset describing the inverse cumulative normal distribution of the proportion of subjects below the reference limit, multiplied by the standard deviation of η = .839 giving the offset = 1.07 for 90% and offset = .57 for 75% reference limits.
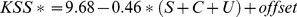
(1.16)


**Figure 5:**
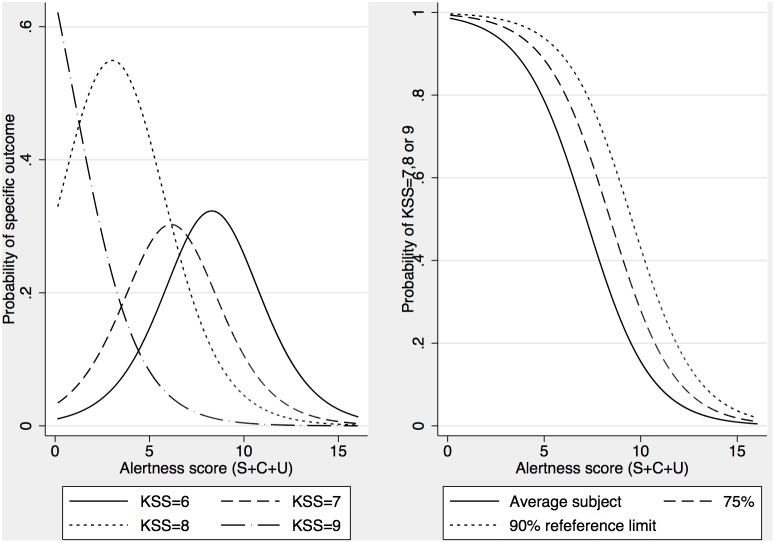
Predicted probabilities as a function of alertness score (S_B_+C+U) based on equation 17. Left panel shows probabilities for specific outcomes (KSS = 6–9) and right panel shows probabilities for severe sleepiness (KSS≥7) with reference limits accounting for 75% and 90% of subjects (below the line) in addition to the average subject (i.e. 50% reference limit).



We also fitted a multilevel mixed effect ordinal logistic regression model (1.14) to estimate the probability of observing any level of sleepiness based on S+C+U. The equation for predicting these probabilities is presented below (equation 17) with cut points estimated at, K = (−10.99, −8.95, −7.67, −6.66, −5.64, −4.30, −3.05, −0.58) for probabilities above KSS>k = (1, 2, 3, 4, 5, 6, 7, 8) respectively and a standard deviation of η  = 1.10, giving offset = 1.41 for 90% and offset = .74 for 75% reference limits. The function is also illustrated in figure 5 showing predictions for specific levels on KSS on the left and probabilities for observing severe sleepiness (KSS≥7) on the right, including the reference limits. Note that probabilities for KSS = 6 initially increase when alertness goes down but decrease when alertness is very low because of increased probability of observing KSS = 7, 8 or 9.

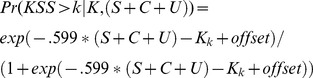
(1.17)


### Exploring circadian type adjustment

**Figure 6:**
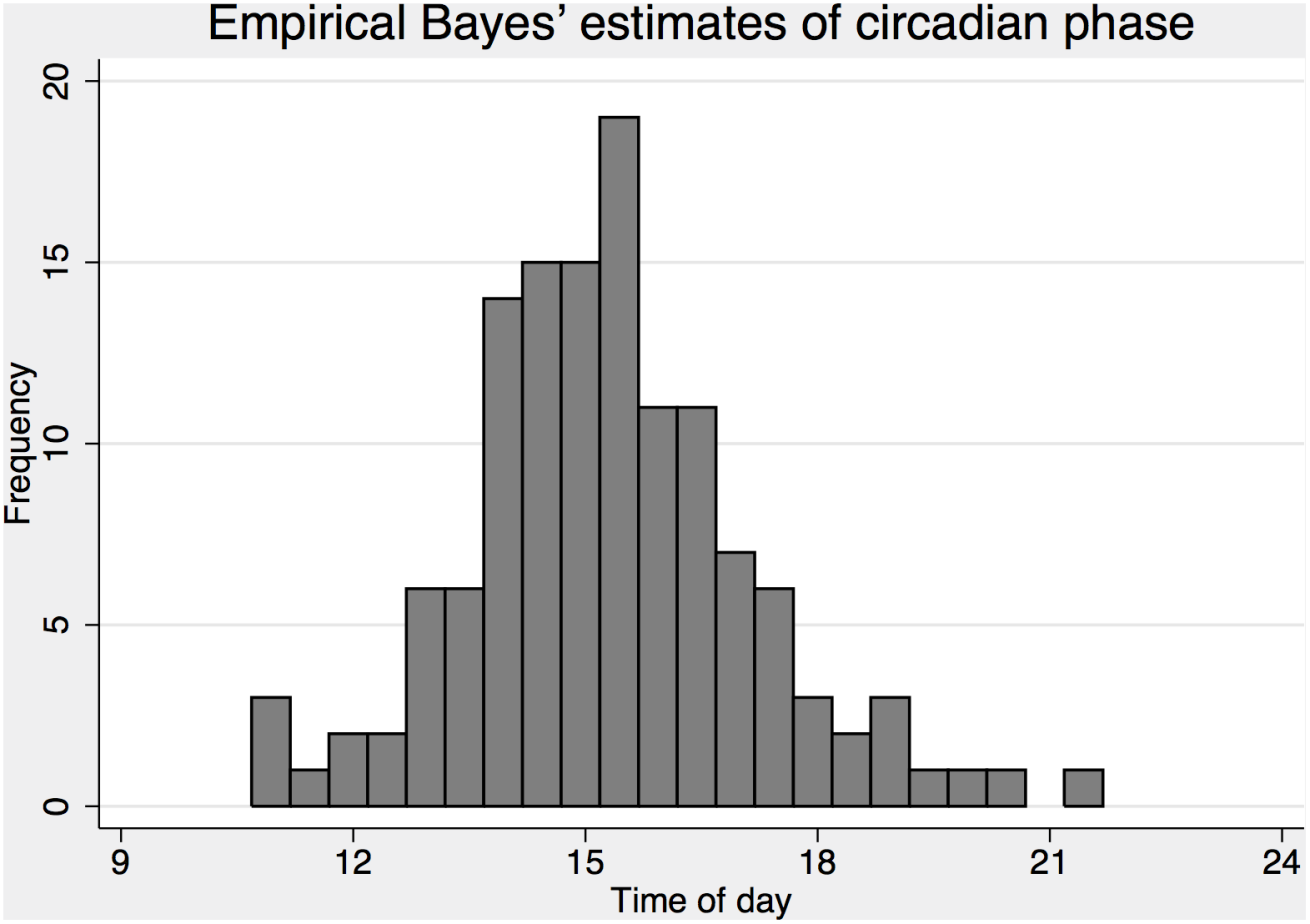
Empirical Bayes’ estimates of all subjects circadian phase based on equation 13.

As a first step towards adjusting the phases of C based of the diurnal type question, we fitted a cosinor analysis to data to empirically determine the phase of C in the data. The first model (1.12) was estimated with a log likelihood of −10107.3, suggesting a large improvement in model fit with a deviance compared to the model with default C (5a) of chi2 = 413. Adding random effects to the model (1.13) provided a highly significant improvement in fit (chi2 = 442, df = 2, p<.001) and made it possible to estimate individual phases of C for all subject. The sine and cosine coefficients were estimated at. 649±.03 (standard error) and. 631±.03, respectively, suggesting a mean phase of p = 15 h in the data with an amplitude of .91 (in the metrics of KSS). The random effects were estimated with standard deviations of.47 and.54 respectively and were used to generate empirical Bayes’ estimates of individual phases of C (figure 6). These phases were subjected to a linear regression model with rated circadian type as the independent variable. The result suggest a significant (p<.001) relation between circadian type and the empirical Bayes estimate of circadian phase with predicted phase of 16.62±.43 h for extreme evening types and −.669±.20 h for each earlier level of the scale corresponding to phases: 14.61 h, 15.28 h, 15.95 h, 16.62 h for the observed circadian type groups (morning type, intermediate type, evening type and extreme evening type).

Predictions with an adjusted phase of C to p = 15 h (6a & 6b) greatly increased fit compared to models with the default phase of C (5a & 5c) with a deviance statistic of chi2 = 358–339. Adding circadian type adjusted phases of C_T_ (based on the circadian type question) further increased the fit of the models with a deviance statistic of chi2 = 88 for a model with S+C_T_ (6d) and chi2 = 89 for a model with S_B_+C_T_+U (6c). The simpler model (6d) with only S_B_+C_T_ provided better fit to data than the model with S_B_+C_T_+U (6c). Residual plots suggest that most of the systematic bias observed for model 5c (figure 3, top panel) was reduced or eliminated for model 6d (figure 3, bottom panels), but evidence of sleep inertia is still visible mainly in the first hour, and there seem to be a slight underestimation of sleepiness at long wake hours and high/low predicted sleepiness, though data is sparse in these areas. The equation for predicting probabilities based on S+C_T_ (model 6d) is presented below (1.18) with cut points estimated at, K = (−11.29, −9.18, −7.82, −6.76, −5.67, −4.27, −3.00, −0.53) for probabilities above KSS>k = (1, 2, 3, 4, 5, 6, 7, 8) respectively and a standard deviation of η  = 1.14, giving offset = 1.46 for 90% and offset = .77 for 75% reference limits. 
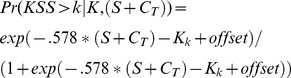
(1.18)


Due to its dependence on the default phase of C (p = 16.8) the sleep generator itself did not perform well with adjusted circadian phases (compared to the standard settings presented above) as indicated by a multilevel mixed effect logistic regression model (1.15) suggesting a conditional probability of pr≈.192 for an observed sleep when no sleep was generated by TPM and pr≈.659 when a sleep was generated and an odds ratio of OR = 8.0 (95% CI: 7.9–8.1).

The observed data suggest that the sleep pattern was dependent on circadian type (figure 4 middle panel) and to address the misfit of sleep patterns we developed a modified sleep generator dependent on only S and C_T_ and tested different thresholds for falling asleep and waking up in 0.5 point steps on the alertness scale. The best result was found with a threshold for falling asleep of S_B_+C_T_<8 and a threshold for awakening of S_B_+C_T_>13. When submitted to a multilevel mixed effect logistic regression model, the conditional probability of observing a sleep was estimated at pr≈.087 when no sleep was generated and increased to pr≈.798 when a sleep was generated and with an odds ratio of OR = 41.4 (95% CI: 40.8–42.1), outperforming the original sleep generator with the default phase presented above (with OR = 28). Figure 4 bottom panel shows observed and generated sleep propensity for all three sleep generators.

The predictions of sleepiness from the model with the new sleep generator (8c) was greatly improved, with a deviance statistics of chi2 = 382, compared to the one using the old thresholds (7a), but the best fit to data was from the model with only process S_B_+C_T_, individual phases and generated sleeps with the new thresholds (8c) with fit statistics that were even better (chi2 = 112) than the original S_B_+C+U model with information of observed sleeps and assuming a single fixed phase at p = 16.8 presented above (5c).

### Exploring acclimatization to different time zones

We fitted models that included the new acclimatization process A (model 11a–11c) on an expanded dataset that also included time zones other than the home base and observed increased fit for models with acclimatization (chi2≈47–57). A model with instant acclimatization (A_100%_) to the new time zone (11a) performed worse than no acclimatization at all (10a).

So far acclimatization has been assumed to equal the default of 50% the difference between acclimatized time and local time suggested by Darwent et al. [45]. To explore a more optimal acclimatization we fitted models with 0–100% adjustments in 5% increments. The results presented in table 3 suggest an optimal rate of ∼30%, with a deviance statistic of chi2≈10 compared to a daily acclimatization of 50% and a deviance of chi2≈57 compared to a model (10a) without acclimatization.

**Table 3:** Model summary of varying daily acclimatization rates.

daily rate	Log likelihood
0%	−15027.5
5%	−15013.5
10%	−15006.5
15%	−15002.4
20%	−15000.3
25%	−14999.3
30%	−14999.1
35%	−14999.6
40%	−15000.6
45%	−15002.0
50%	−15003.8
55%	−15005.8
60%	−15008.2
65%	−15010.9
70%	−15013.9
75%	−15017.1
80%	−15020.7
85%	−15024.7
90%	−15029.5
95%	−15035.7
100%	−15086.0

## Discussion

### Main findings

The present study has validated the Three Process Model of alertness (TPM), including extensions added since its inception in 1990. The result suggests that with an assumed default phase of 16.8 h for process C, an optimal model includes the processes S_B_+C+U but not W. However, with an improved and circadian type adjusted C (C_T_), process U is no longer part of the model, questioning the validity of the ultradian component. We could also validate the added predictive power of the “brake function” that slows down process S_B_ by making it linear for most of the sleep period. Model based generated sleeps are feasible if observed sleeps are not available but with significantly increased error in predictions. Acclimatization of process C_T_ to a different time zone was also possible, but with an optimal rate of ∼30% instead of the assumed daily rate of 50% of the difference between local time and internal acclimatized time. In addition, the TPM was extended with reference limits to describe the influence of individual differences in sleepiness propensity as well as a function to provide probabilities of any level of sleepiness for assessment of risk in safety applications.

### Limitations

Before the results are discussed in detail some limitations of the findings should be discussed. One limitation of this study is the use of an application to collect data that has not been previously validated against any independent source. The work and sleep timing, background information (such as age, gender, position) and circadian type, were assessed based on face validity, but the sleepiness ratings was made using an established subjective sleepiness scale (KSS) transferred to iOS software platform. We have little reason to believe that this has had any negative effects on the quality of the data compared to other established methods (i.e. using pen and paper versions of validated scales) but believe that the extensive validation of input performed by the application (see methods section) may have reduced the error in reported sleep and work timings.

Another main limitation of the present study is the crowd sourcing based data collection and self-selection of participants. There is no guarantee that participating individuals were who they claimed to be (i.e. captains, first officers and cabin crew in airline operations). However, the tailor made application for air crew (CrewAlert) and the procedure to specifically target airline crew by advertising on conferences and through official channels in airlines and unions together with the survey code attached to data submitted for analysis, does increase the likelihood that participants were active in airline operations. We cannot claim the study population to be representative of all airline operations. However, the main mechanisms of sleep regulation (the homeostatic process “S” and circadian process “C”) studied in the present study is present in all healthy humans suggesting, at least, a relatively high internal validity for the model to predict sleepiness. We also focused our analyses only on data contained in custom sleep logs, which reduced the number of participants from 153 to 136. We believe this decision was necessary to secure independent data for analysis and avoid circular estimation.

### Validation of the original TPM

The best-fitted model included processes S_B_+C+U with the “brake function” (i.e. S_B_) but residual plots indicate some systematic bias in the model and explorative analyses suggested (see below) that the phase of C was not optimal and that U should not be part of the model. We could not validate that the sleep inertia function (process W) adds to the model. This is probably because sleep inertia is mainly in effect after forced awakening, with a substantial partial sleep deprivation, and such situations were not common in the present data. The residual analysis suggests that an inertia function would fit the data if it were much smaller in magnitude and it is possible that an adaptive function that depends on sleep pressure at awakening would have been more appropriate, but exploring such possibility was outside of the scope of the present study.

### Extending the model with probabilities and individual differences for ecological risk estimates

We used multilevel mixed effect logistic models to account for individual differences and provide functions for probabilities of all levels of sleepiness based on the alertness score generated by TPM. Unlike earlier models developed to predict group means [30], these functions can provide un-biased estimates in the presence individual differences of non-linear effects and report metrics useful for applications to calculate absolute and relative risks of any level of sleepiness for individual work shifts, shift sequences and whole schedules. The reported functions also add the ability to assess risks accounting for a specified proportion of subjects (e.g. 50%, 75% and 90%) that may be used to adapt risk estimates to varying safety requirements. To our knowledge, this is a unique quality in sleepiness/fatigue modeling and we know of no other models with similar features. For example, neurobiological performance modeling is usually based on modeling mean data aggregated at the group level [48], which might lead to bias in the estimated parameters of non-linear effects in the presence of individual differences [8], [49], and provides no information about individual variability around the mean (or median) predictions.

### Exploring circadian type adjustment and a new phase of C

Explorative analyses indicate that the default phase of process C (p = 16.8 h) was not optimal and suggested a phase ∼1.8 h earlier (p≈15 h) than default. The reason for this discrepancy is not clear but there are a few possibilities. We have to consider bias in the estimates due to sampling (very few samples at night). The relative lack of data at night can be seen as a missing data problem and the models used in the present study are robust against missing data as long as it is missing at random (MAR). This assumption means that missing data will not bias estimates as long as the model can predict the probability of missing data as a function of alertness and this should reduce the potential bias to a minimum in the present study.

Another possibility is that aircrew might constitute a selection of individuals with more morning tendency than the average individual. However, our data show that this is an unlikely explanation since there was a relatively even distribution of evening-morning types. The linear prediction of individual phases also suggests that even extreme evening types would have an average phase earlier (p≈16.6) than the assumed default phase (p = 16.8), although the data for this analysis was sparse (only 2 subjects rated themselves as extreme evening types) and the estimated range of phases illustrated in figure 6 does suggest some individuals with phases as late as p≈20–21 h. It should be recognized that we used empirical Bayes’ estimates (also known as BLUP, Best Linear Unbiased Predictors, in linear models) to assess individual phases. Such estimates are shrunken towards the group mean, with more shrinkage in extreme individuals and few observed data points, to reduce error of the individual subject estimate. Thus, estimates of circadian phases were optimized for unbiased predictions of individuals, but may represent a conservative estimate of the true variance in the group. Also, we did not use individually determined phases when we fed this information back to our model for prediction of sleepiness; instead, we used the estimated group average for different circadian types. This would make model adjustments conservative for the more extreme individuals within each circadian type.

A potential problem with the TPM-model is that the shape of the circadian rhythm could be poorly approximated by a cosine function. For example, if the trough of the circadian rhythm would be flatter, a sleepiness peak would be observed significantly later because of increased sleepiness due to time awake (process S) over time. With the standard cosine function, such an increase in S is immediately countered by a steep rise in C. It is also possible that sleepiness peaks in studies where individuals are awake all night may be affected by a simultaneous shift in the circadian phase. Such delayed phase after nighttime wakefulness/sleep deprivation has been demonstrated in laboratory conditions [50] and would suggest a momentary delaying of the phase before, and likely also a partial advancing, after, the trough, mainly as a result of light exposure during the sensitive part of the phase response curve [51], [52]. Such mechanism has, to our knowledge, never been explicitly studied in a real life situation but seems plausible given that normal indoor lighting seems to be sufficient to shift the phase [52] and that there is an observed tendency to phase delay sleep and sleepiness patterns after working night shifts [53], [54]. Our data showed limited exposure to full night shifts so the circadian phase would mainly be estimated on non-night work where the phase seems to be stable at an earlier hour.

Individual empirical Bayes’ estimates of circadian phases were validated against the circadian type question and by adjusting the models according to rated circadian type with a large increase in model fit. The best models with adjusted phases of C_T_ did not include process U questioning the validity of a 12 h-ultradian component in sleepiness regulation. The results suggest that process U was part of the model only to adjust some of the misfit from the original process C. This finding suggests that the main mechanisms of sleep and sleepiness regulation could be reduced to the original two processes, S and C, and likely also with a modified (adaptive) process W as discussed above.

### Exploring a new model based sleep generator

The original sleep generator did not work well with the adjusted phases (C_T_) due to its dependency on the default phase of C. But the new sleep generator developed in the present study provided better predictions of sleep than the original generator. The improvement of the sleep generator was so large that the new model *without* knowledge of sleep even outperformed the original model *with* knowledge of sleep in predicting sleepiness (the best model over-all, however, was the new model with knowledge of observed sleep timings). This suggests a large potential improvement in evaluating work schedules where information of sleep times may not be available. However, it is possible that the sleep generator developed in the present study is specific to this group and such modifications need to be validated on other data and tested for feasibility in different situations. Also, other work on sleep prediction suggests that sleep timing may be the effect of two circadian processes: one is the endogenous rhythm (i.e. process C_T_) and the other is an exogenous social rhythm [45]. The latter is particularly important for sleep when traveling to different time zones since the social rhythm is instantaneously shifted to local time without the need for acclimatization. The sleep-generating algorithm in TPM is based on a compromise between these two rhythms.

### Exploring acclimatization to different time zones (jetlag) with process A

We could validate the feasibility of acclimatization to different time zones with the new process A, both after returning home from a different time zone with jetlag and in a more general setting when flying east or west. These results give strength to the conclusion that the TPM could be improved by a dynamic adjustment to different time zones for aircrew and travelers. The optimal daily adjustment rate seems to be slower than has been previously been suggested [45] and was found to be ∼30% in the present study.

### Conclusions

The present study has validated the internal processes of TPM on aircrew and explored potential large improvements to the parameters and sleep generator based on a question of circadian type. We have also extended the model to include individual differences, reference limits accounting for 50%, 75% and 90% of the population as well as a direct prediction of probabilities of any level of sleepiness for absolute and relative risk assessment of work schedules in safety applications. The explorative findings and extension made to the model need further validations in independent studies, ideally with large representative samples to provide normative data on model parameters.
